# Supplementary material for: Data-driven discovery of potent small molecule ice recrystallisation inhibitors
Source: Nat Commun. 2024 Sep 15;15:8082. doi: 10.1038/s41467-024-52266-w (PMC11402961; doi:10.1038/s41467-024-52266-w)
Supplement: Supplementary file 1 — Supplementary Information [file 41467_2024_52266_MOESM1_ESM.pdf]

Data-driven Discovery of Potent Small Molecule Ice Recrystallisation Inhibitors Supplementary Information

Matthew T. Warren[1,2,3], Caroline I. Biggs[1], Akalabya Bissoyi[2,4,5], Matthew I. Gibson[1,2,4,5*] and Gabriele C. Sosso[1*]

[1]Department of Chemistry, University of Warwick, Gibbett Hill Road, Coventry, CV4 7AL, United Kingdom.
[2]Warwick Medical School, University of Warwick, Gibbett Hill Road, Coventry, CV4 7AL, United Kingdom.
[3]Current address: Department of Biochemistry, University of Oxford, South Parks Road, Oxford, OX1 3QU, United Kingdom.
[4]Current address: Department of Chemistry, University of Manchester, Oxford Road, Manchester, M13 7PL, United Kingdom.
[5]Manchester Institute of Biotechnology, University of Manchester, 131 Princes Street, Manchester, M1 7DN, United Kingdom.

*Corresponding author(s). E-mail(s): m.i.gibson@warwick.ac.uk; g.sosso@warwick.ac.uk;

1 Supplementary Discussions

1.1 Splat cooling assay conditions

As discussed in the main text, it is essential to include saline (or other additives) in the solution when performing the "splat cooling" assay, to ensure a eutectic phase is formed and allow for ice recrystallisation to occur; false positives can arise when using pure water or low concentrations of solutes for these measurements. Considering this, a solution such as phosphate buffered saline (PBS) is typically employed (with ∼140 mM NaCl). However, this concentration of saline is not essential and relevant IRI data has been obtained in lower salt concentrations when using materials which are not stable or soluble in PBS [1, 2].

In this work, a 10 mM NaCl solution is used for all IRI activity measurements (i.e. all compounds in the *Amino* dataset), as in our previous work [3, 4]. Compounds were assessed at 20 mM, unless otherwise stated. However, for the *Glyco* and *Glyco2* datasets, measurements were performed in PBS. Consequently, % MGS values obtained may not always be directly comparable, with crystals typically appearing larger in assays performed with PBS. To investigate the effects of these conditions, we performed a cross-comparison of % MGS values obtained using 10 mM NaCl and PBS for a selection of compounds in the *Glyco(2)* dataset (Supplementary Figure S1). Given that the aim of this work to predict IRI activity of novel molecules in saline, this data suggests that it is possible to train models using the data obtained under different conditions.

1.2 Prediction set accuracy

We observed that % MGS predictions for active molecules (% MGS < 20) in the prediction set were often higher than the experimental measurements. This is a consequence of averaging across many individual models to obtain a consensus. While individual descriptor models are capable of making predictions at the extremes of the % MGS distribution (i.e. close to 0 and 100 % MGS), the process of averaging means these values are less likely to be observed for ensemble predictions. In our prediction library, ensemble predictions with low % MGS values were also excluded from the prediction set due to high prediction uncertainty.

1.3 Splat cooling assay measurement confidence and error

We acknowledge the measurements obtained using the "splat cooling" assay have inherent noise and variability arising from a number of sources, including the initial number/crystal size which results from a stochastic nucleation process. We also note that larger errors are typically observed for weaker materials, as exemplified in Figure 2a. The choice of this technique was motivated by experimental throughput, as well as a desire to compare against other materials reported in literature, where this technique is widely used [1, 2, 5–9].

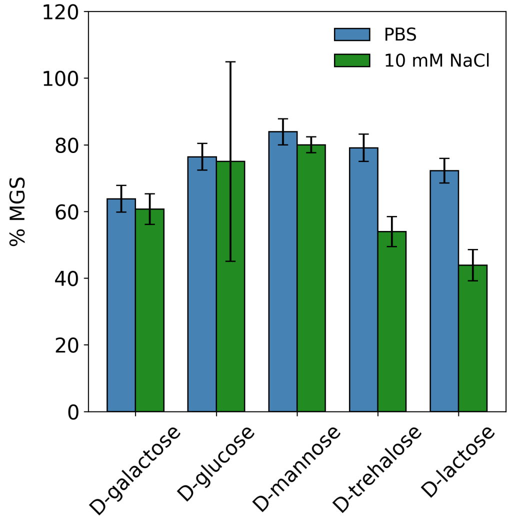

Fig. S1: IRI activities for a selection of sugars in the *Glyco(2)* dataset tested at 22 mM using the "splat cooling" assay in PBS or NaCl. Bars show the mean +/- one SD from three independent repeats.

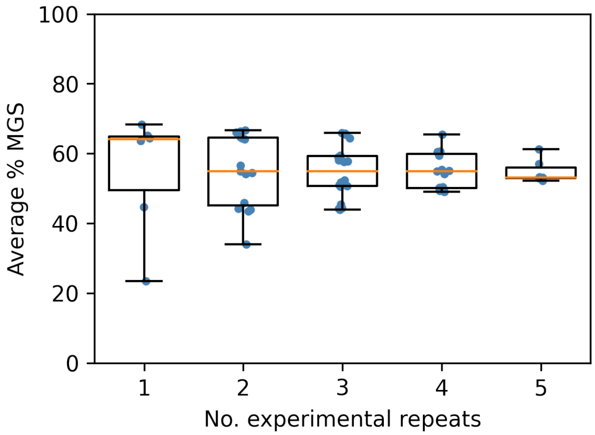

Fig. S2: Distributions of average % MGS values from a retrospective analysis of measurements obtained using the "splat cooling" assay for L-alpha-alanine. Given six % MGS measurements, we computed the average % MGS using one to five repeats by taking the mean of all possible combinations of the six measurements. Using all six measurements gave an average % MGS = 54.4. The boxes show the interquartile range and median (orange line) of the data.

2 Supplementary Methods

2.1 Experimental Methods

2.1.1 Nanolitre osmometry

The morphologies of ice crystals during their growth and melting were analysed using an Otago nanoliter osmometer (Otago Osmometers, Dunedin, New Zealand), as previously described [10]. A temperature-controlled stage with an oil-filled sample well was employed, and a drop of analyte measuring approximately 100 mm in diameter (equivalent to 0.5 nl) was injected into the well. The stage was then cooled until the drop nucleated and froze. Subsequently, the stage was gradually warmed to melt the sample, leaving only single ice crystal behind. This remaining ice crystal was subjected to multiple cycles of heating and cooling at the desired temperature to investigate its shaping behaviour. The observations were captured with a Canon EOS 1200D digital SLR camera and an Olympus CX41 microscope outfitted with a UIS 20x/0.45/*/0-2/FN22 lens.

2.1.2 Red blood cell cryopreservation

For all red blood cell (RBC) freezing experiments a solution was prepared using a 30% glycerol solution, diluted from a commercially available glycerol solution with 0.2% dextrose and 0.9% saline as per a previously established protocol [11]. This freezing solution was added in an equal volume to 150 μL RBC suspension, resulting in a final volume of 300μL. To ensure uniform ice nucleation at a sub-zero temperature of -5 °C, the RBC suspensions were carefully transferred to 96-well plate and allowed to incubate at room temperature for 10 min prior to the controlled nucleation process.

A standardised 5-minute hold time was implemented to guarantee consistent nucleation. Subsequently, the samples were gradually cooled at a rate of 1 °C per minute until reaching a temperature of -40 °C, followed by immediate thawing through immersion in a 37 °C water bath. Post-thaw haemolysis was assessed using the ADH method [12]. These controlled freezing conditions were replicated between 4 times (n=3) for each freezing solution, with particular emphasis on evaluating the impact of two key compounds, namely, 2-Amino-4-methylthiazole-5-carboxylic acid (Compound **7**) and 4,4-dimethylpyrrolidine-2-carboxylic acid (**16**) across five different concentrations: 110 mM, 55 mM, 25 mM, 12.5 mM, 6.25 mM and 3.12 mM. This comprehensive approach allowed for a thorough investigation into the cryoprotective properties of these compounds on RBC preservation.

Following the transient warming procedure described in Ref. [11], RBC suspensions were transferred to cryotubes, left to incubate at room temperature for 10 minutes, and then immersed in dry ice (-80 °C). Continuous temperature monitoring using a 1 second interval temperature probe guided the subsequent steps. After reaching -80 ± 2 °C, the samples were immersed in a methanol bath cooled to -20 °C, followed by another plunge into dry ice once the internal

solution reaches -20 °C. RBC samples were kept in dry ice until the internal solution, as monitored by the temperature probe, reached -80 ± 2 °C. At this point, samples were either thawed (representing one cycle of transient warming) or re-immersed in a methanol bath cooled to -20 °C. The experiment included one, three, and five cycles of immersion in a -20 °C methanol bath and dry ice. Thawing was expedited by plunging samples into a 37 °C water bath. For post-thaw analysis, the AHD assay was used as described in the following section. These conditions were replicated three times (n=3) for each freezing solution.

All RBC samples were derived from sterile sheep blood purchased from Tebubio Ltd.

2.1.3 Haemolysis assay

The haemolysis assay procedure followed a meticulously designed series of steps aimed at evaluating the impact of cryoprotectants (CPAs) on haemolysis rates. Initially, a concentrated cryoprotectant solution was prepared, with adjustments made for pelleting if necessary. Two distinct 10 mL blood samples were placed in centrifuge tubes and subjected to a brief 5-minute spin at 2000 rpm to isolate RBCs. After centrifugation, the pale supernatant devoid of RBCs, typically measuring 6-7 mL, was carefully discarded. These blood samples were then replenished with 7 mL of Dulbecco's Phosphate-Buffered Saline (DPBS) each and gently mixed through inversion. Subsequently, 150 μL of blood was combined with an equal volume of cryoprotectant solution (CPA) within a cryovials, with DPBS serving as a control. In cases involving pellet formation, 1x CPA was added to a pellet derived from 300 μL of blood. Following a 10-minute incubation, a controlled freezing process was initiated, gradually cooling the samples at a rate of -1 °C per minute until reaching -40 °C, facilitated by a cooler. Thawing was conducted either within a 37 °C water bath for 5 minutes or at room temperature.

For analysis, two Eppendorf tubes were assigned per cryovial: one for receiving the thawed sample for subsequent centrifugation and the other for mixing with AHD. A precise 100 μL of the thawed sample was transferred to the first Eppendorf tube, spun for 5 minutes, and 40 μL of supernatant were collected. This supernatant was then mixed with 750 μL of AHD in the second Eppendorf tube, vigorously vortexed before adding 200 μL of the sample into three separate wells for each condition, totalling nine for each type of CPA. The plate was analysed at 580 nm to assess haemolysis rates, providing insights into the cryoprotectants' efficacy in preserving red blood cell integrity. Recovery was calculated as: (1-(Absorbance-DPBS/(lysate-DPBS)) $\times$ 100 [12]

2.2 Computational Methods

2.2.1 Classification metrics

Sensitivity, which is also known as recall or the true positive rate (TPR), represents the proportion of positive instances (i.e. active compounds) that are correctly identified among all the positive predictions:

$$\text{Sensitivity} = \frac{\text{TP}}{\text{TP} + \text{FN}} \tag{1}$$

where TP represents the number of true positives (active compounds correctly predicted as active) and FN represents the number of false negatives (active compounds incorrectly predicted as inactive). Precision, on the other hand, provides insight into the number of truly active compounds identified in terms of the total number of active predictions:

$$\text{Precision} = \frac{\text{TP}}{\text{TP} + \text{FP}} \tag{2}$$

where FP represents the number of false positives (inactive compounds incorrectly predicted as active). Consequently, there is always a trade-off between the sensitivity and precision of a model. This can be quantified using the F-score, which represents the harmonic mean of the two quantities and provides an overall assessment of the classifier's accuracy:

$$\text{F} = 2 \cdot \frac{\text{Sensitivity} \cdot \text{Precision}}{\text{Sensitivity} + \text{Precision}} \tag{3}$$

The final classification metric featured in this work is specificity, or the true negative rate (TNR), which represents the proportion of negative instances (i.e. inactive compounds) that are correctly predicted among all the negative predictions:

$$\text{Specificity} = \frac{\text{TN}}{\text{TN} + \text{FP}} \tag{4}$$

Thus, whilst perfect sensitivity and specificity are theoretically possible, in reality a classification model must be optimised to achieve a balance between these terms, depending on the specific application.

2.2.2 Correlations between IRI activity and molecular size

The Pearson correlation coefficient (PCC) for molecular volumes and IRI activities are -0.23 and -0.45 for the *Glyco2* and *Amino* datasets, respectively.

2.2.3 Molecular descriptor parameters

Supplementary Table 1 - SOAP descriptor parameters used for each dataset. Parameters were optimised using a genetic algorithm described in Ref. [13]

Dataset	$n^{\max}$	$l^{\max}$	Cutoff	Sigma
Glyco(2)	7	8	7	0.38
Amino	6	9	2	0.67
Combined	7	5	5	0.90

Supplementary Table 2 - H-wACSF descriptor parameters used for each dataset.

Dataset	N^{rad}	N^{ang}	R_c^{rad} (Å)	R_c^{ang} (Å)	N_{bins}
Glyco(2)	8	10	10	12	10
Amino	10	16	10	20	8
Combined	8	10	10	12	10

2.2.4 Neural network hyperparameters

For classification models, the output layer for each model consisted of a single node and sigmoid activation function. For regressors, the output layer comprised a single node with a linear activation function. Batch sizes of 16 and 32 were used to train all *Amino* and *Glyco(2)* models, respectively.

Supplementary Table 3 - Neural network classifier hyperparameters used for *Glyco* dataset.

Model	Activation function	Optimiser	Learning rate	Hidden layers
Standard descriptors	ReLu	Adagrad	0.01	40-20-10
Molecular cliques	ReLu	Adagrad	0.01	40-20-10
H-wACSFs	ReLu	Adagrad	0.01	100-100-100
SOAPs	ReLu	Adadelta	0.01	100-100-100
Hydration histograms	ReLu	Adagrad	0.01	30-30-30
Hydration indices	ReLu	Adagrad	0.01	30-30-30

2.2.5 Maximum expected performance

The maximum expected performance (i.e. maximum correlation or minimum error) of predictive models trained using the *Amino* dataset was estimated using the method described by Brown et al. [14]. Briefly, simulated data was first generated by adding an error to the target value for each datapoint in the *Amino* dataset. The errors were sampled from a Gaussian distribution defined by a mean of 0 and standard deviation equal to the (mean) standard deviation

Supplementary Table 4 - Neural network regressor hyperparameters used for *Glyco2* dataset.

Model	Activation function	Optimiser	Learning rate	Hidden layers
Standard descriptors	ReLu	Adagrad	0.01	60-60-60
Molecular cliques	ReLu	RMSprop	0.01	30-30-30
H-wACSFs	ReLu	Adagrad	0.01	60-60-60
SOAPs	ReLu	Adagrad	0.01	100-100-100
Hydration histograms	ReLu	Adam	0.01	60-30-10
Hydration indices	ReLu	Adam	0.01	10-10-10

Supplementary Table 5 - Neural network regressor hyperparameters used for *Amino* dataset.

Model	Activation function	Optimiser	Learning rate	Hidden layers
Standard descriptors	ReLu	Adagrad	0.005	20-40-80
Molecular cliques	ReLu	RMSprop	0.01	60-60-60
H-wACSFs	ReLu	Adagrad	0.01	60-60-60
SOAPs	ReLu	Adagrad	0.01	100-100-100
Hydration histograms	ReLu	RMSprop	0.01	10-10-10
Hydration indices	ReLu	RMSprop	0.01	50-50-50

associated with the experimental measurements for the *Amino* dataset (11.8 % MGS units). The Pearson correlation coefficient (PCC) and mean squared error (MSE) values of the simulated and true data were then calculated. This process was repeated 1000 times, resulting in an distribution of PCC and MSE values (Supplementary Figure S3) from which the maximum expected performance can be estimated.

2.2.6 Repeated leave-one-out cross validation

Model performance for repeated leave-one-out cross validation is shown in Supplementary Tables 6 and 7.

Supplementary Table 6 - Performance metrics for test predictions obtained from regression models evaluated using a repeated leave-one-out cross validation procedure. Results shown are the means across five repeats ± one standard deviation.

Dataset	Model	MSE	PCC
Glyco2	Std. descriptors	637 ± 19	0.377 ± 0.03
Amino	Std. descriptors	724 ± 37	0.551 ± 0.03

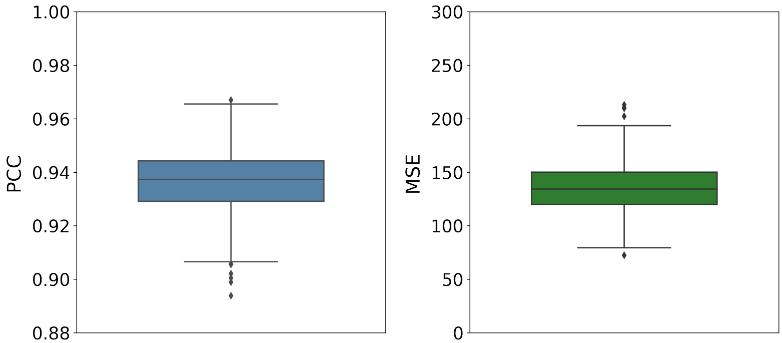

Fig. S3: Distribution of maximum expected Pearson correlation coeffient (PCC, left) and mean squared error (MSE, right) metrics achievable for the a predictive model trained using the *Amino* dataset. PCC and MSE values were calculated from 1000 trials using simulated data, as described in the Supplementary Information.

Supplementary Table 7 - Performance metrics for test predictions obtained from classification models trained on the *Glyco* dataset, evaluated using a repeated leave-one-out cross validation procedure. Results shown are the means across five repeats ± one standard deviation.

Model	Sensitivity	Specificity	Precision	F-score
Std. descriptors	0.570 ± 0.02	0.659 ± 0.01	0.750 ± 0.01	0.647 ± 0.01

2.2.7 Tanimoto similarities

In the context of cheminformatics, the Tanimoto similarity coefficient quantifies the similarity between two chemical compounds based on the presence or absence of specific structural features encoded as fingerprints. It is computed as the intersection (number of common features) divided by the union (total number of features) of the two sets of features, providing a value between 0 (no similarity) and 1 (complete similarity). In this work, Tanimoto similarities were calculated for the ECFP4 fingerprints or Molecular ACCess System (MACCS) keys. All calculations were performed using RDKit [15].

2.2.8 Dimensionality reduction

Prior to performing dimensionality reduction, the descriptor features were standardised by subtracting the mean and dividing by the standard deviation of the features (i.e. Z-score normalisation). Principal component analysis (PCA) was performed using scikit-learn [16], which computes the principal components via singular value decomposition (SVD) on the covariance matrix

of the descriptor features. t-Distributed stochastic neighbour embedding (t-SNE) was also performed using the implementation in scikit-learn. A perplexity value of 20 was used to preserve local and global structure of the data, alongside the default learning rate of 200.

3 Supplementary Figures

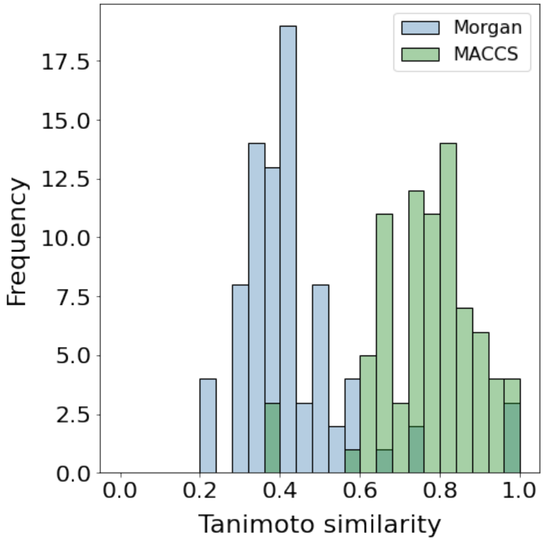

Fig. S4: Distribution of the Tanimoto similarities for compounds added to the *Glyco2* dataset, compared to the *Glyco* dataset. Tanimoto similarities were determined for two fingerprints – ECFP4 and Molecular ACCess System (MACCS) – computed via RDKit. For each compound, only the highest similarity score is shown. Values of 1 indicate isomeric (as opposed to identical) structures, which are indistinguishable based on their ECFP4/MACCS fingerprints.

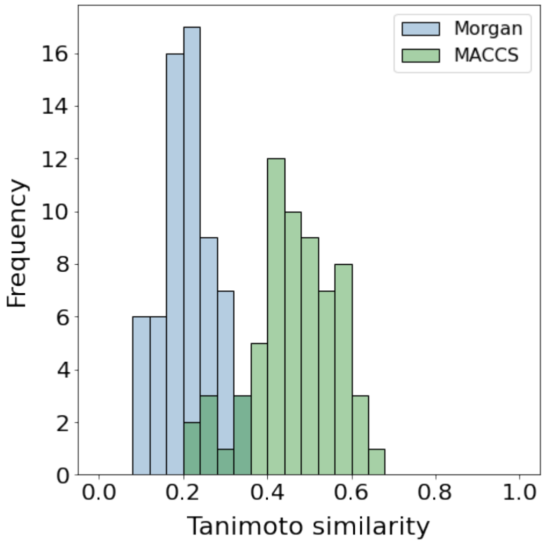

Fig. S5: Distribution of the Tanimoto similarities for compounds in the *Amino* dataset, compared to the *Glyco2* dataset. Tanimoto similarities were determined for two fingerprints – ECFP4 and Molecular ACCess System (MACCS) – computed via RDKit. For each compound, only the highest similarity score is shown.

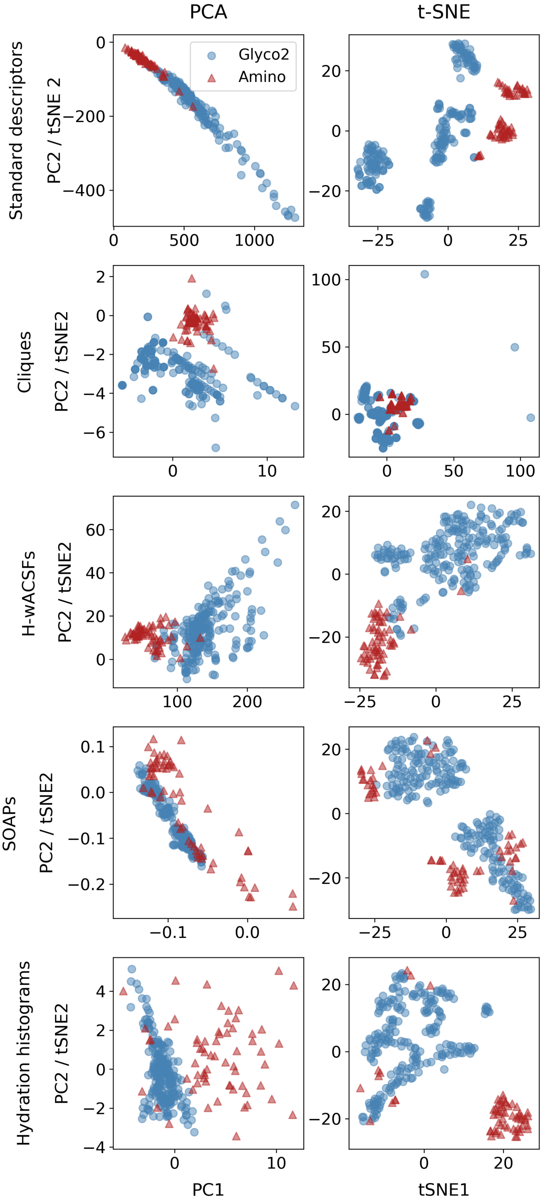

Fig. S6: Principal component analysis (PCA) and t-distributed stochastic neighbour embedding performed on the descriptor features for the *Glyco2* (blue circles) and *Amino* (red triangles) datasets. From top to bottom: standard descriptors, molecular cliques, H-wACSFs, SOAPs and hydration histograms.

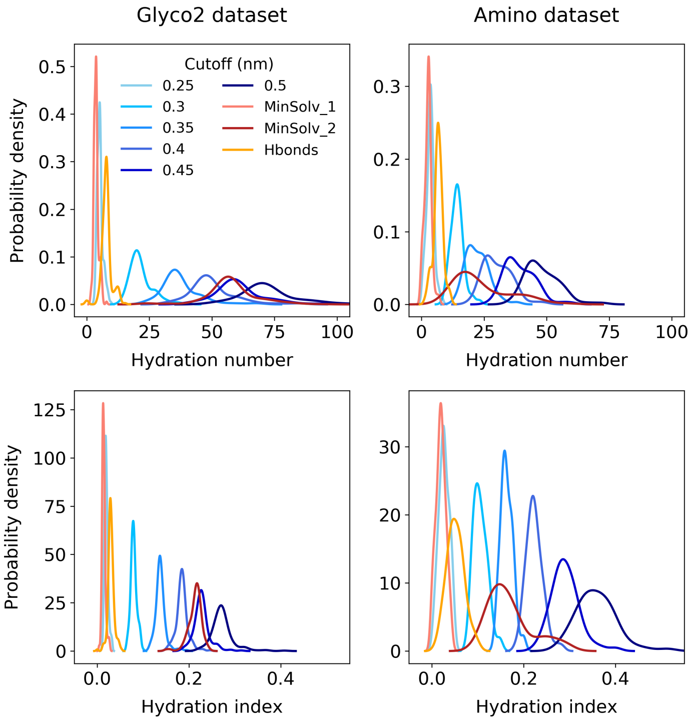

Fig. S7: Probability distributions for hydration numbers (top) and hydration indices (bottom) for *Glyco2* (left) and *Amino* (right) datasets.

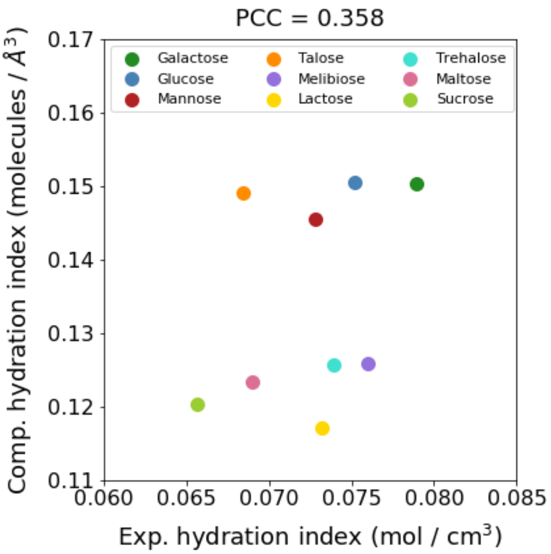

Fig. S8: Scatter plot showing correlation between computational hydration indices calculated using the method described here and experimental hydration indices reported by Tam et al. [6] for a set of nine mono- and disaccharides.

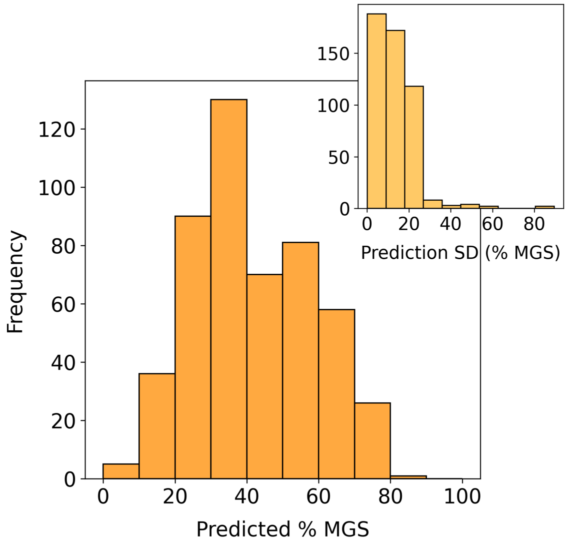

Fig. S9: Frequency histograms showing the distribution of predicted % MGS values for ∼500 compounds in the prediction library, obtained using an ensemble model. Inset shows the distribution of error (SD) for the predictions using the different descriptors included in the ensemble.

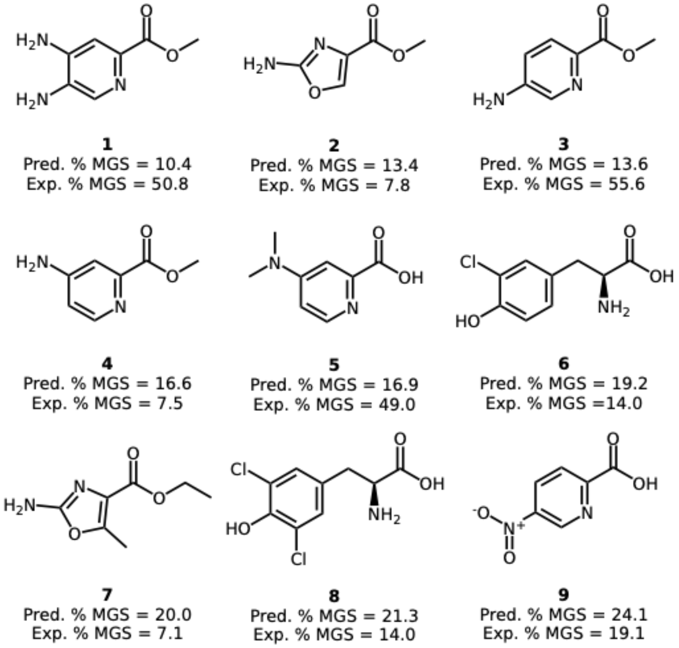

Fig. S10: Chemical structures and IRI activities of "active" predictions. IRI activity was measured at 20 mM in 10 mM NaCl using the "splat cooling" assay.

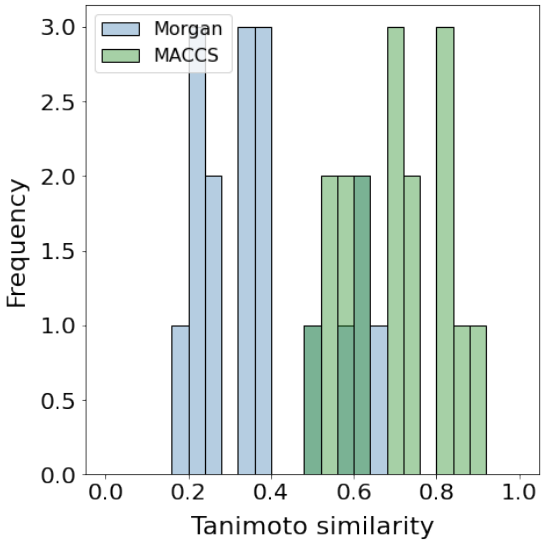

Fig. S11: Distribution of the Tanimoto similarities for compounds in the *Amino* prediction set, compared to the *Amino* training dataset. Tanimoto similarities were determined for two fingerprints – Extended-connectivity fingerprints (ECFP4) and Molecular ACCess System (MACCS) – computed via RDKit. For each compound, only the highest similarity score is shown.

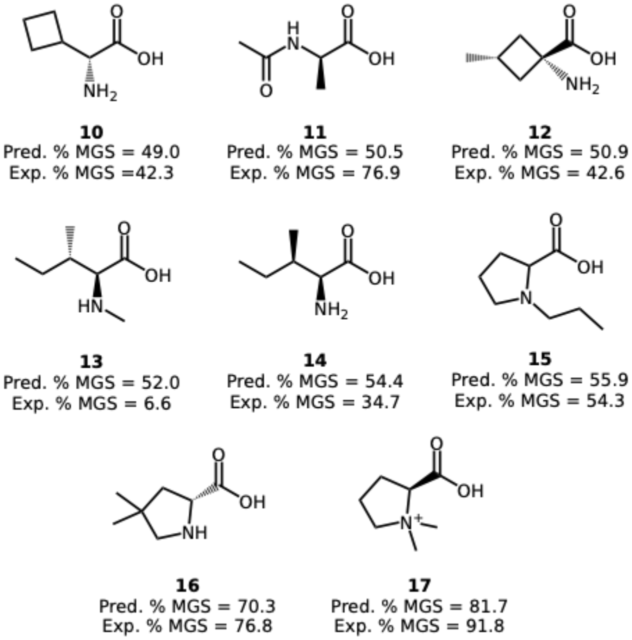

Fig. S12: Chemical structures and IRI activities of "inactive" predictions. IRI activity was measured at an amino acid concentration of 20 mM in 10 mM NaCl using the "splat cooling" assay.

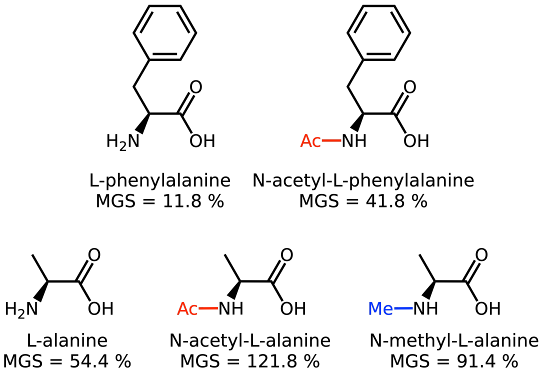

Fig. S13: Chemical structures and IRI activities of N-modified L-phenylalanine and L-alanine. IRI activity was measured at an amino acid concentration of 20 mM in 10 mM NaCl using the "splat cooling" assay.

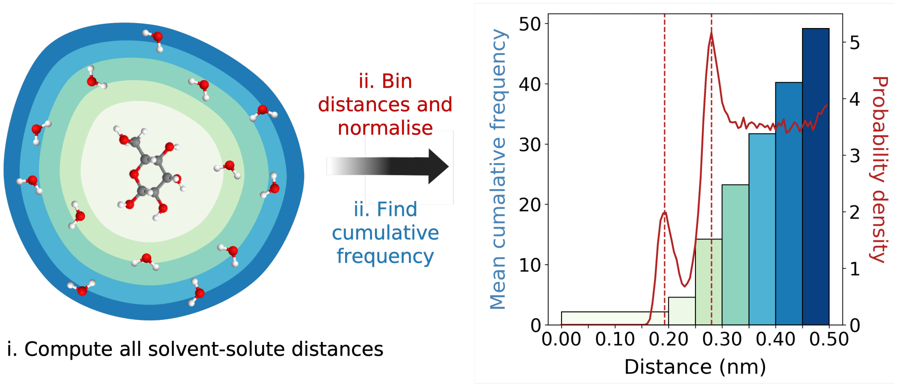

Fig. S14: Construction of hydration histograms and calculation of hydration numbers. In this illustration, mean cumulative frequencies (i.e. hydration numbers) have been obtained using seven cutoffs from 0.20 to 0.50 in 0.05 nm intervals. Solid red line shows the probability density histogram computed as outlined in the Methods. Dashed red lines show the peaks corresponding to the first and second solvation shells. Only the oxygen atoms in solvent molecules were considered when computing solvent-solute distances.

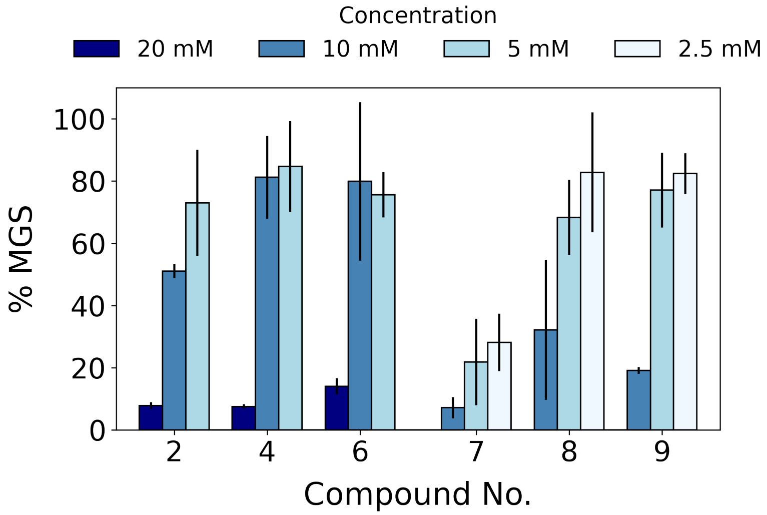

Fig. S15: IRI activities of amino acids predicted to be active. IRI activity was determined using the "splat cooling" assay using the amino acid concentration shown, in 10 mM NaCl. Bars show the mean +/- one SD from three independent repeats.

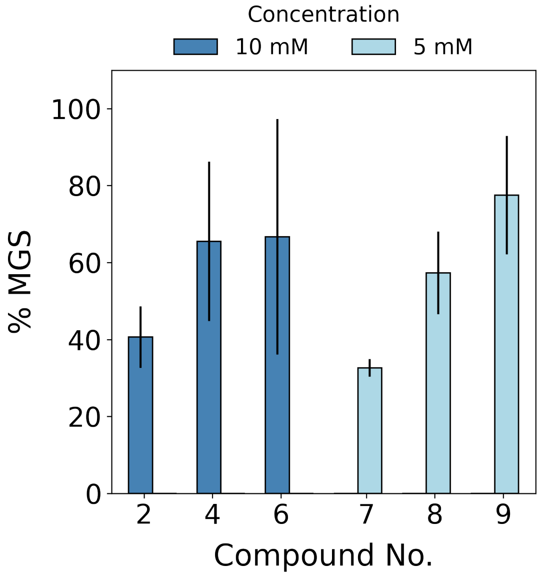

Fig. S16: IRI activities of amino acids predicted to be active. IRI activity was determined using the "splat cooling" assay in phosphate-buffered saline (PBS). Bars show the mean +/- one SD from three independent repeats.

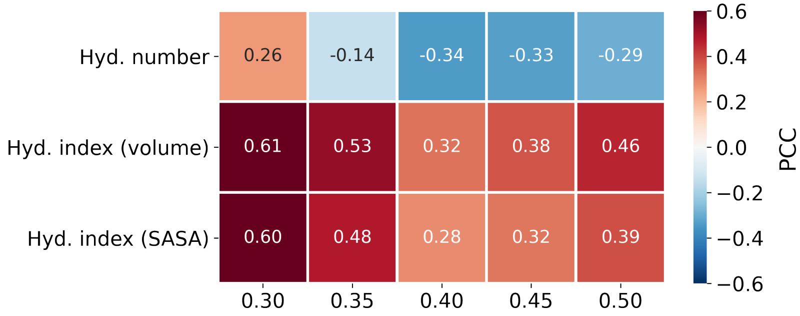

Fig. S17: Heatmap showing the correlation (PCC) between % MGS values and hydration numbers or hydration indices calculated using different cutoffs and size metrics for the *Amino* prediction set. Hydration indices were obtained by dividing the hydration numbers by either the molecular volume or SASA, as indicated.

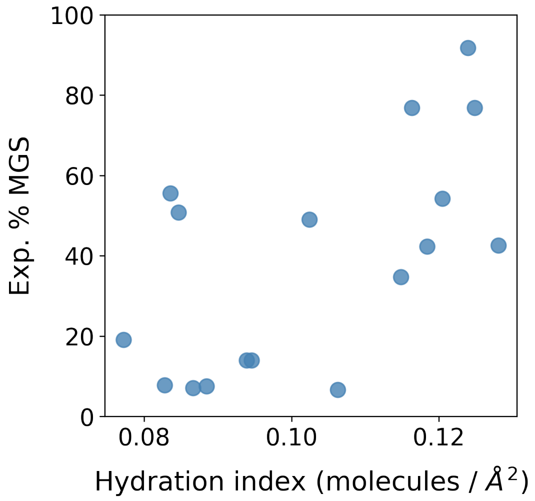

Fig. S18: Scatter plot showing the correlation between hydration indices calculated for the prediction set and % MGS. Hydration numbers were determined using a cutoff of 0.3 nm and normalised by solvent-accessible surface area, as described in Methods.

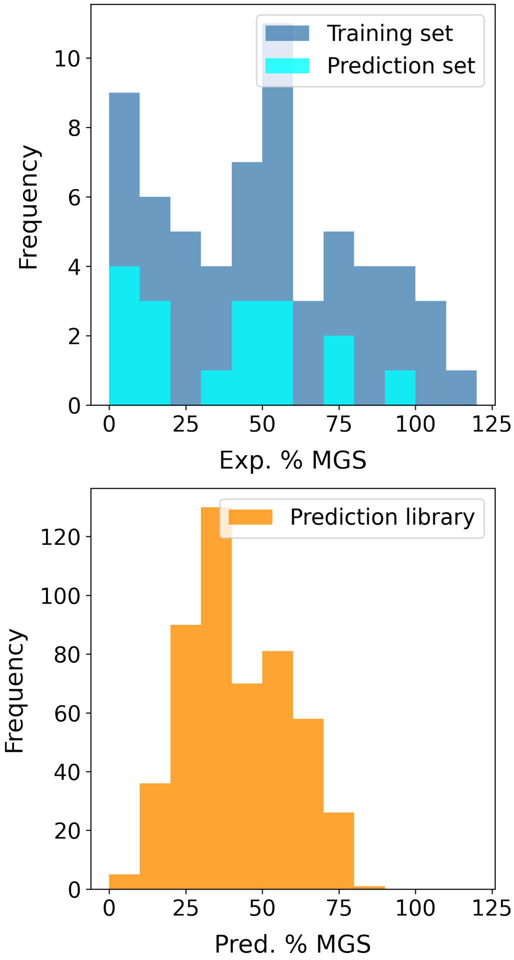

Fig. S19: Histograms showing the distribution of experimentally determined % MGS values for the Amino training and prediction sets (top), and predicted % MGS values for the Amino prediction library obtained from the ensemble model.

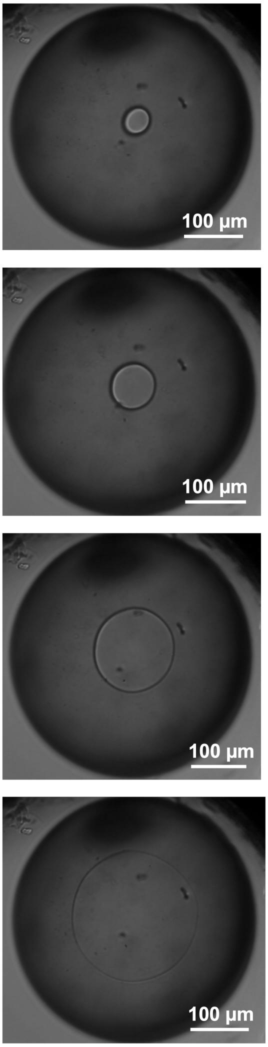

Fig. S20: Micrographs from single crystal ice shaping assay peformed on ethyl 2-amino-5-methyl-1,3-oxazole-4-carboxylate (compound **7**) at 5 mM.

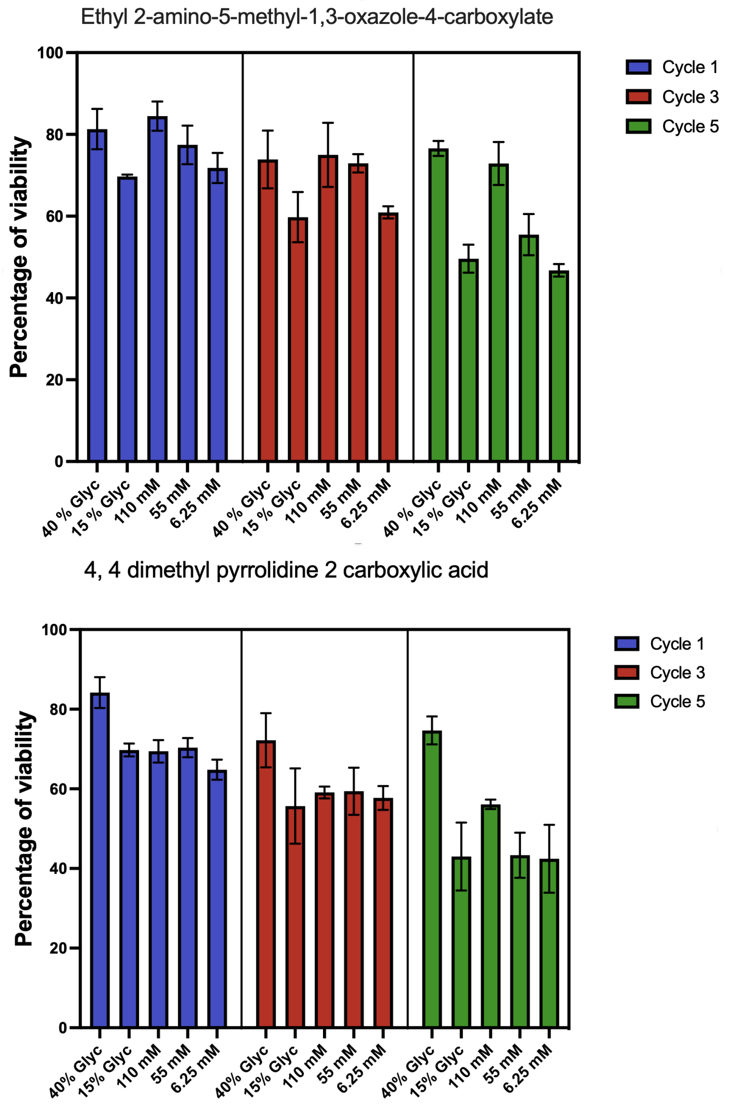

Fig. S21: Percentage of intact RBCs after transient warming injury utilising different cryosolutions: 40% glycerol, 15% glycerol, and 15% glycerol with either 110 mM, 55 mM, or 6.25 mM of ethyl 2-amino-5-methyl-1,3-oxazole-4-carboxylate (compound **7**, top) and 4,4-dimethylpyrrolidine-2-carboxylic acid (compound **16**, bottom) respectively.

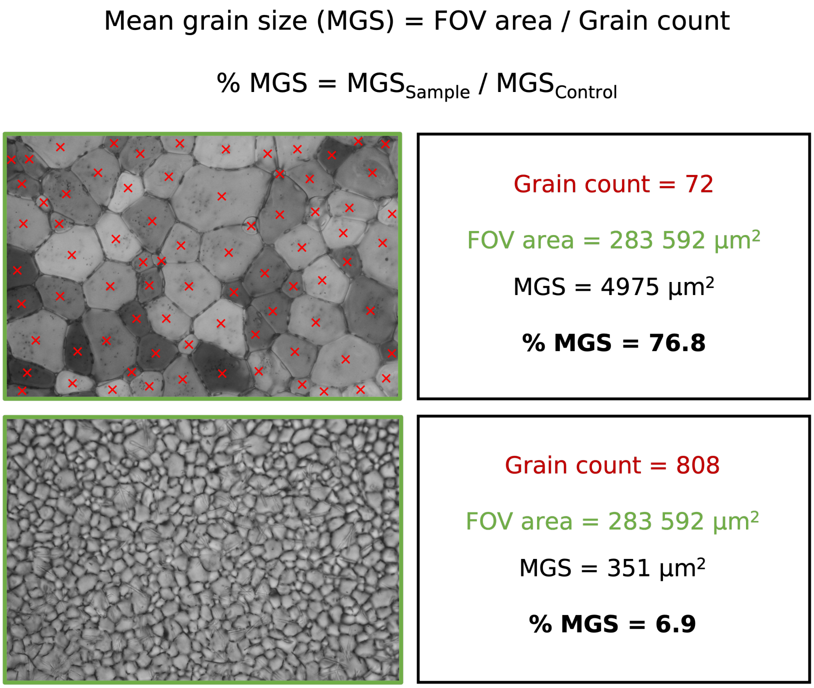

Fig. S22: Procedure for calculating % MGS from crystal micrographs obtained using the "splat cooling" assay. The images for two representative micrographs for an active (bottom) and inactive (top) compound are shown, revealing the entire field of view (FOV). The number of ice grains are first counted, and the FOV area is divided by this number to obtain the mean grain size (MGS). MGS values are normalised against a positive control for ice growth (i.e. 10 mM saline) yielding a % MGS value.

References

[1] Georgiou, P.G., Marton, H.L., Baker, A.N., Congdon, T.R., Whale, T.F., Gibson, M.I.: Polymer Self-Assembly Induced Enhancement of Ice Recrystallization Inhibition. Journal of the American Chemical Society **143**(19), 7449–7461 (2021). https://doi.org/10.1021/jacs.1c01963

[2] Balcerzak, A.K., Febbraro, M., Ben, R.N.: The importance of hydrophobic moieties in ice recrystallization inhibitors. RSC Advances **3**(10), 3232–3236 (2013). https://doi.org/10.1039/c3ra23220d

[3] Warren, M.T., Galpin, I., Bachtiger, F., Gibson, M.I., Sosso, G.C.: Ice Recrystallization Inhibition by Amino Acids: The Curious Case of Alpha- and Beta-Alanine. The Journal of Physical Chemistry Letters **13**, 2237–2244 (2022). https://doi.org/10.1021/acs.jpclett.1c04080

[4] Warren, M.T., Galpin, I., Hasan, M., Hindmarsh, S.A., Padrnos, J.D., Edwards-Gayle, C., Mathers, R.T., Adams, D.J., Sosso, G.C., Gibson, M.I.: Minimalistic ice recrystallisation inhibitors based on phenylalanine. Chemical Communications **58**(55), 7658–7661 (2022). https://doi.org/10.1039/d2cc02531k

[5] Knight, C.A., Hallett, J., DeVries, A.L.: Solute effects on ice recrystallization: An assessment technique. Cryobiology **25**(1), 55–60 (1988). https://doi.org/10.1016/0011-2240(88)90020-X

[6] Tam, R.Y., Ferreira, S.S., Czechura, P., Ben, R.N., Chaytor, J.L.: Hydration index-a better parameter for explaining small molecule hydration in inhibition of ice recrystallization. Journal of the American Chemical Society **130**(51), 17494–17501 (2008). https://doi.org/10.1021/ja806284x

[7] Capicciotti, C.J., Kurach, J.D.R., Turner, T.R., Mancini, R.S., Acker, J.P., Ben, R.N.: Small molecule ice recrystallization inhibitors enable freezing of human red blood cells with reduced glycerol concentrations. Scientific Reports **5** (2015). https://doi.org/10.1038/srep09692

[8] Briard, J.G., Fernandez, M., De Luna, P., Woo, T.K., Ben, R.N.: QSAR Accelerated Discovery of Potent Ice Recrystallization Inhibitors. Scientific Reports **6**(1), 26403 (2016)

[9] Briard, J.G., Jahan, S., Chandran, P., Allan, D., Pineault, N., Ben, R.N.: Small-Molecule Ice Recrystallization Inhibitors Improve the Post-Thaw Function of Hematopoietic Stem and Progenitor Cells. ACS Omega **1**(5), 1010–1018 (2016). https://doi.org/10.1021/acsomega.6b00178

[10] Judge, N., Georgiou, P.G., Bissoyi, A., Ahmad, A., Heise, A., Gibson, M.I.: High molecular weight polyproline as a potential biosourced ice

growth inhibitor: synthesis, ice recrystallization inhibition, and specific ice face binding. Biomacromolecules (2023)

[11] Briard, J.G., Poisson, J.S., Turner, T.R., Capicciotti, C.J., Acker, J.P., Ben, R.N.: Small molecule ice recrystallization inhibitors mitigate red blood cell lysis during freezing, transient warming and thawing. Scientific Reports **6**(2319) (2016). https://doi.org/10.1038/srep23619

[12] Red Blood Cell Cryopreservation with Minimal Post-Thaw Lysis Enabled by a Synergistic Combination of a Cryoprotecting Polyampholyte with DMSO/Trehalose. Biomacromolecules **23**(2), 467–477 (2022)

[13] Barnard, T., Hagan, H., Tseng, S., Sosso, G.C.: Less may be more: An informed reflection on molecular descriptors for drug design and discovery. Molecular Systems Design and Engineering **5**(1), 317–329 (2020). https://doi.org/10.1039/c9me00109c

[14] Brown, S.P., Muchmore, S.W., Hajduk, P.J.: Healthy skepticism: assessing realistic model performance. Drug discovery today **14**(7-8), 420–427 (2009)

[15] Landrum, G.: Rdkit: Open-source cheminformatics software (2016)

[16] Pedregosa, F., Varoquaux, G., Gramfort, A., Michel, V., Thirion, B., Grisel, O., Blondel, M., Prettenhofer, P., Weiss, R., Dubourg, V., Vanderplas, J., Passos, A., Cournapeau, D., Brucher, M., Perrot, M., Édouard Duchesnay: Scikit-learn: Machine learning in python. Journal of Machine Learning Research **12**(85), 2825–2830 (2011)
